# Inhibition of Acute Lung Injury by TNFR-Fc through Regulation of an Inflammation-Oxidative Stress Pathway

**DOI:** 10.1371/journal.pone.0151672

**Published:** 2016-03-18

**Authors:** Yuan Weifeng, Li Li, Hu Yujie, Li Weifeng, Guo Zhenhui, Huang Wenjie

**Affiliations:** 1 Department of Respiratory Medicine, General Hospital of Guangzhou Military Command of PLA, Guangzhou, Guangdong, China; 2 Department of Respiratory Medicine, Hospital No 458 of PLA, Guangzhou, Guangdong, China; 3 MICU, Key Laboratory of Geriatric Infection and Organ Function Support of Guangdong Provincial, General Hospital of Guangzhou Military Command of PLA, Guangzhou, Guangdong, China; University of North Dakota, UNITED STATES

## Abstract

**Background:**

Acute lung injury (ALI), characterized by disruption of the lung alveolar-capillary membrane barrier and resultant pulmonary edema, and associated with a proteinaceous alveolar exudate, is a leading cause of morbidity and mortality. Currently, inflammation-oxidative stress interaction between TNF-α and NF-κB was identified as a key pathway of ALI. We hypothesized that a TNFR-Fc fusion protein would have beneficial effects in experimental ALI, and sought to test this idea in mice by blocking TNF-α.

**Methods and Results:**

Intratracheal instillation of lipopolysaccharide (LPS) into the lungs of ALI mice led to histiocyte apoptosis, and detection of serum and bronchoalveolar lavage fluid (BALF) cytokines, feedback between NF-κB and TNF-α, lung albumin leakage, lung damage, IκB kinase (IKK) and NF-κB activation, I-κB degradation, and oxidative injury. LPS administration raised pulmonary inflammation as reflected by increased inflammatory cytokines, alveoli protein concentration, and ALI scores. IKK is phosphorylated following LPS challenge, leading to I-κB degradation and NF-κB p65 phosphorylation. Furthermore, NF-κB is translocated into the nucleus and up-regulates TNF-α gene transcription. Infusion of TNFR-Fc 24h before LPS challenge significantly abrogated the increase of inflammatory cytokines, especially serum TNF-α concentration, as well as pulmonary alveoli protein levels, and diminished IKK and NF-κB activation and I-κB degradation. The nuclear translocation of NF-κB was inhibited, following by down-regulation of TNF-α gene transcription. In addition, LPS intratracheal instillation induced marked oxidative damage, such as a decrease in total anti-oxidation products and an increase in malondialdehyde (MDA), as well as up-regulation of oxidation enzymes. Histologic analysis and apoptosis scores revealed that the extent of tissue lesions was significantly reduced, but not abrogated, by TNF-α blockade.

**Conclusion:**

Treatment with LPS alone increased inflammation and oxidative stress in ALI mice, while administration of TNFR-Fc 24h before LPS challenge broke the feedback between NF-κB and TNF-α, resulting in decreased pulmonary inflammation/oxidative damage and tissue destruction. These results suggest a potential role for TNF-α therapy to treat clinical ALI.

## Introduction

Tumor necrosis factor alpha (TNF-α) is a multifunctional cytokine that participates in the pathophysiology of the systemic inflammatory response in critically ill patients. Acute respiratory distress syndrome (ARDS) is one disease mediated by TNF-α [1]. Acute lung injury (ALI) is the early stage of ARDS that may reside within the therapeutic window of ARDS. ALI involves pulmonary edema, macromolecules, and inflammatory cells migrating into the bronchoalveolar compartment. In ALI patients, TNF-α levels are elevated in both the serum and the bronchoalveolar lavage fluid (BALF) [2, 3], and administration of TNF-α produces endotoxic shock [4], a pathological process similar to ALI. Therefore, anti-TNF-α antibodies have been used to protect against sepsis-associated lethality [5, 6].

Because of the beneficial effects of blocking TNF-α in inflammatory disorders, many pharmaceutical companies have started to explore drugs that block TNF-α. Etanercept is a recombinant protein constructed by fusing human soluble p75 TNF receptors (extracellular domain) to human IgG1 Fc (TNFR-Fc), and has been used to ameliorate rheumatoid arthritis symptoms [7] and ankylosing spondylitis [8].

Although the beneficial effect of TNFR-Fc in patients with rheumatoid arthritis or other chronic inflammatory diseases are well documented, it is not clear whether TNFR-Fc ameliorates acute inflammatory diseases, such as ALI. In this study, we used TNFR-Fc in mice to block TNF-α, which is endogenously generated following intratracheal LPS administration. Intratracheal LPS administration induces acute inflammation and oxidative damage in the lung. Pretreatment of TNFR-Fc resulted in a significant reduction of proinflammatory cytokines and pulmonary vascular leakage. In addition, TNFR-Fc interrupts inflammation—oxidative stress feedback by inhibiting kinase activation and NF-κB nuclear translocation. These data provide initial evidence that TNFR-Fc may be a feasible interventional approach for ALI treatment.

## Materials and Methods

### Animals

This study was carried out in strict accordance with the recommendations in the Guide for the Care and Use of Laboratory Animals. The protocol was approved by the Institutional Animal Care and Use Committee (IACUC) at Guangzhou General Hospital of Guangzhou Military Command (Guangzhou, China). All surgery was performed under sodium pentobarbital anesthesia, and all efforts were made to minimize suffering.

BALB/c mice (weighing 20–25 g) were purchased from Medical Laboratory Animals Center of Guangdong (Foshan, China). Mice were housed for a minimum of 5 days in a specific pathogen-free facility with access to food and water in a temperature-controlled room with a 12 h light/dark cycle.

Animals were allocated to 3 groups, as follows: 1) control, 2) LPS intratracheal administered, 3) TNFR-Fc intraperitoneal administered + LPS intratracheal administered. To generate ALI mouse models, LPS (E. coli O55:B5, 5mg/Kg, Sigma, St. Louis, MO, USA) was intratracheally administered in 50 μL saline. For the TNFR-Fc + LPS group, TNFR-Fc (0.4mg/Kg) was given intraperitoneally in 50 μL saline followed by LPS treatment after 24 hours. Animals were sacrificed 2 h after LPS/saline treatment.

### Measurement of Cytokines and Protein

Blood plasma samples were centrifuged at 4°C (Beckman-Coulter, Brea, CA, USA) at 2,500 rpm for 15 min and used for estimating circulating TNF-α, IL-6, and IL-10. BALF was performed with 2 ml PBS. BAL fluid was centrifuged at 1,000g for 10 min to obtain cell-free BAL fluid. Serum TNF-α, IL-6, and IL-10 concentration and BAL fluid were quantified using ELISA (Wuhan Boster Bio-Engineering Limited Company, Wuhan, China). BAL protein concentration was measured using a BCA Protein Assay kit (Beyotime Biotech, Jiangsu, China).

### Histopathology

Lungs were perfused free of blood with PBS and fixed at 20 cm H2O with 4% paraformaldehyde. After fixation, lungs were embedded in paraffin, cut into 2 μm sections for histological evaluation, and stained by hematoxylin and eosin as described previously [9].

### Western Blot Analysis

Phosphorylated IKK and I-κB were by assessed by western blot analysis. Plasma extracts were prepared from lung tissue using Cytoplasmic and Nuclear Protein Extraction Kit (Wuhan Boster Bio-Engineering Limited), and centrifuged at 12,000g for 10 min. Protein extract concentration was estimated with a BCA Protein Assay kit (Beyotime Biotech). Equal amounts of protein (25 μg/lane) were resolved by 8% SDS-PAGE, and transferred onto a PVDF membrane. The membrane was then washed with TBST and blocked in TBST containing 5% non-fat dried milk, and further incubated with the respective specific antibodies to p-IKKα/β, IKKα, IKKβ, p-I-κB, I-κB, p-NF-κB p65 and NF-κB p65, and β-actin. Membranes were then incubated with appropriate HRP-conjugated secondary antibodies, and developed in ECL Western detection reagents (Millipore, Billerica, MA, USA). All experiments were repeated three times.

### Electrophoretic Mobility Shift Assay (EMSA)

The binding of nuclear NF-κB to the TNF-α promoter was assessed by EMSA. Nuclear extracts were prepared from lung tissue using Cytoplasmic and Nuclear Protein Extraction Kit (Wuhan Boster Bio-Engineering Limited), and centrifuged at 14,000 rpm for 10 min at 4°C. Supernatant was stored at -80°C until used for EMSA. NF-κB-specific oligonucleotide 5′- AGGGGGCTTTCCCT -3′ from the murine TNF-α gene promoter was synthesized (TaKaRa, Mountainview, CA, USA). DNA probes were labeled with biotin, using Biotin 3' End DNA Labeling Kit (Pierce Biotechnology, Rockford, IL, USA). Equal amounts of nuclear protein were incubated with biotin-labeled NF-κB oligonucleotide probe for 30 min and run on 5% PAGE at 100 V. The gel and nylon membranes were layered as a sandwich in a clean electrophoretic transfer unit and transferred at 100V for 30 minutes. Transferred DNA was cross-linked to membranes with a hand-held UV (254 nm) for 5–10 minutes, and biotin-labeled NF-κB probe detected by chemiluminescence.

### TNF-α and Oxidative Enzyme Transcription Levels

Lung total RNA was prepared using TRIzol Reagent (Invitrogen, Carlsbad, CA, USA) and diluted to 500 ng/μL. For quantitative real-time polymerase chain reaction (qRT-PCR) analysis of mRNA, cDNA was prepared by reverse transcription. cDNAs were used in all qRT-PCR reactions using SYBR® PrimeScript® RT-PCR Kit II (TaKaRa). Oligonucleotide primers and annealing temperatures used are provided in S1 Table. Annealing temperature dictated by the other genes being amplified in the same reaction.

### Oxidative Damage Assay

Lung was harvested after all mice had been sacrificed and homogenized in RIPA buffer on ice, and centrifuged at 12,000g for 10 min at 4°C and the supernatant collected. Tissue proteins were diluted to appropriate concentrations and subjected to malondialdehyde (MDA) analysis and Total Antioxidant Capacity (T-AOC) assay following the manufacturer’s instructions (Beyotime Biotech, Jiangsu, China). MDA levels were detected by Multiskan MK3 Multi-Mode Microplate Readers (Thermo Fisher, USA) at 532 nm, while T-AOC was measured at 420 nm.

### Statistical Analysis

Values are expressed as the mean ± SEM. A one-way analysis of variance (ANOVA) followed by LSD testing was applied to assess the statistical significance of the differences among the study groups. A value of *P* < 0.05 was considered statistically significant.

## Results

### TNFR-Fc Decreased LPS-Induced Lung Destruction

**Fig 1:**
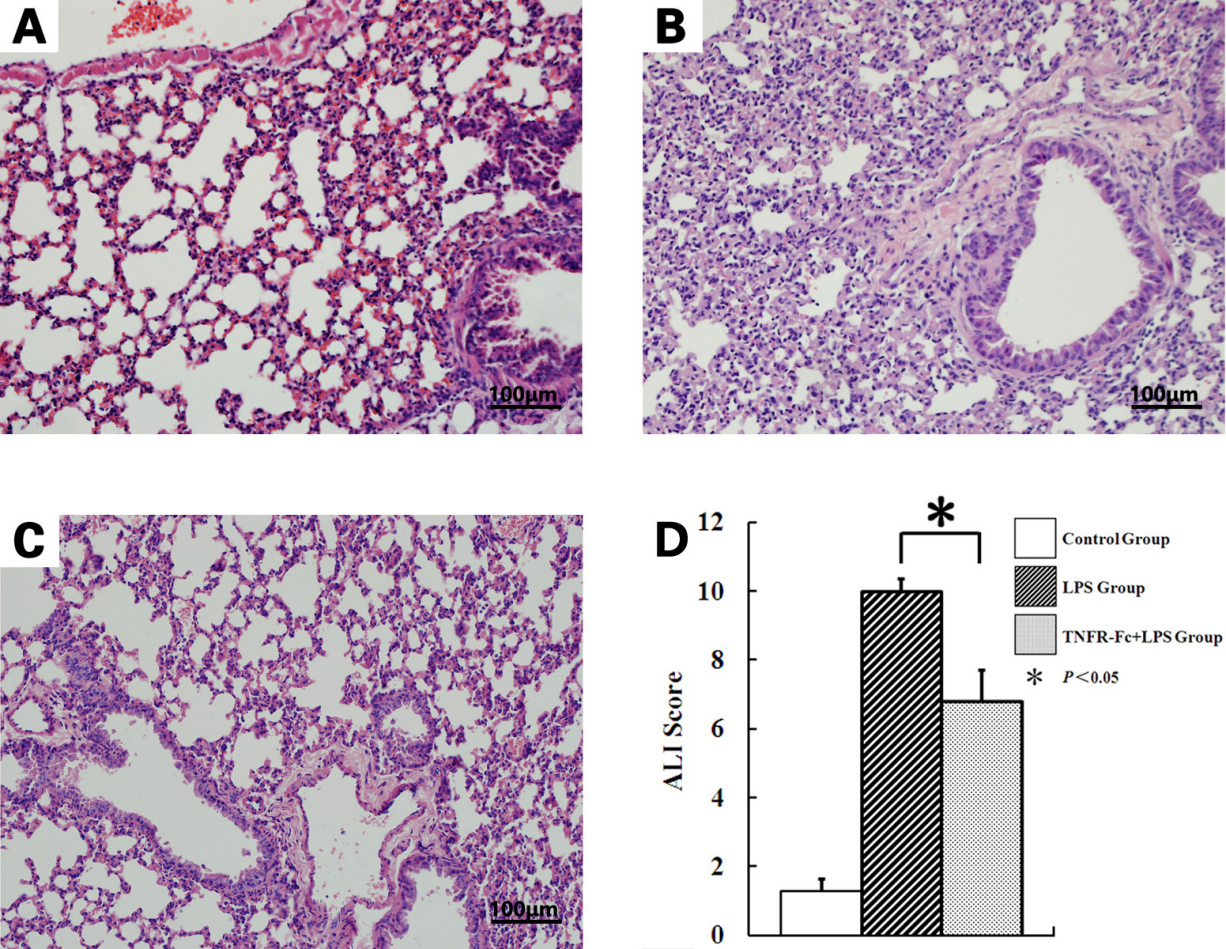
TNFR-Fc attenuates lung destruction induced by LPS injection (x200). Representative hematoxylin and eosin-stained preparations of lung tissue from mice 2 h after LPS/saline treatment (N = 3 in each group). (A) Saline-treated mice display regular lung histology. (B) LPS-treated mice displayed typical signs of congestion and disruption of alveolar architecture. (C) TNFR-Fc-pretreated ALI mice were largely protected from these alterations. (D)Histological assessment of the effect TNFR-Fc on LPS-induced lung inflammation and injury (N = 3 in each group). TNFR-Fc pretreatment decreased the ALI score in ALI mice.

LPS and TNFR-Fc+LPS treated lungs were not qualitatively different from those from the control group, in which no inflammatory changes were observed. However, the alveolar walls showed destructive changes after LPS instillation, marked by interstitial edema and vascular congestion. TNFR-Fc+LPS group The ALI score was significantly lower in mice treated with TNFR-Fc + LPS than those treated with LPS alone (10.0±0.365, 6.8±0.901, *P <* 0.05) (Fig 1).

### Cytokine Concentration

**Fig 2:**
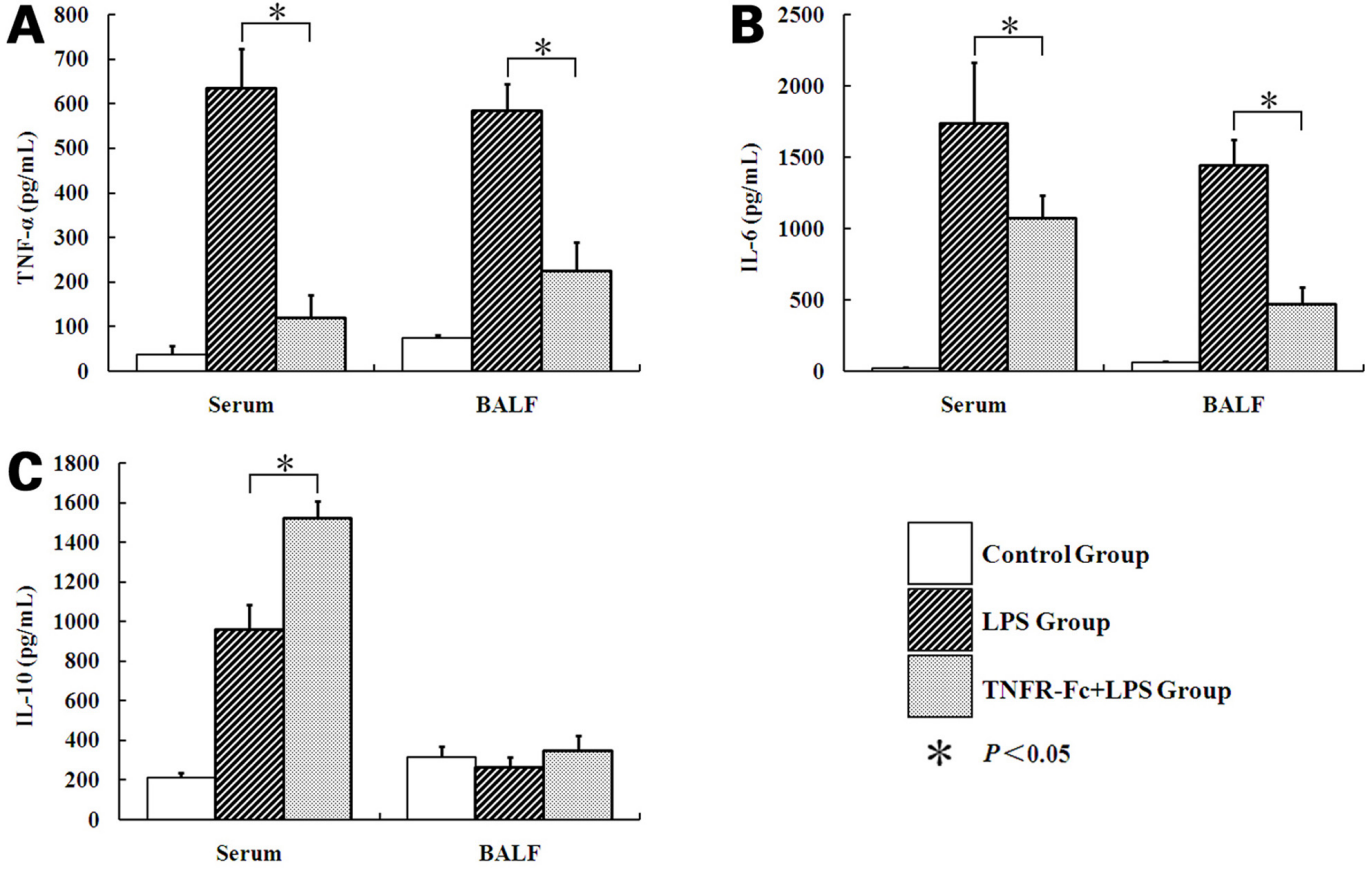
Concentration of TNF-α, IL-6, and IL-10 in serum and BALF after LPS/saline injection. Increase in TNF-α and IL-6 from baseline in serum/BALF obtained from mice in response to LPS or TNFR-Fc + LPS. TNFR-Fc decreased serum/BALF TNF-α and IL-6 levels in ALI mice. Serum IL-10 concentration increased after LPS injection. TNFR-Fc increased serum IL-10 in ALI mice. (A) TNF-α, (B) IL-6, (C) IL-10. Error bars represent SEM (N = 3 in each group). *P < 0.05.

ALI mice demonstrated significant increases in serum/BALF TNF-α over baseline after LPS challenge. At this point, LPS mice had a fivefold increase compared with TNFR-Fc+LPS mice in serum and a 2.6 fold increase in BALF (serum: 634.90±89.082, 119.40±50.874, *P* < 0.05; BALF: 584.16±59.733, 225±63.351, *P <* 0.05). In addition, IL-6 was elevated after LPS injection in ALI mice. Interestingly, mice pretreated with TNFR-Fc had a lower serum/BALF IL-6 level after LPS challenge (serum: 1739.03±423.206, 1073.48±156.062, *P <* 0.05; BALF: 1440.14±184.585, 468.37±123.615, *P <* 0.05). TNFR-Fc pretreatment also increased serum IL-10 levels in ALI mice (958.13±125.345, 1522.52±86.189, *P <* 0.05), but BALF IL-10 concentration was distinct from serum. All groups had detectable IL-10 in BALF but there were no significant differences among them (Fig 2).

### TNFR-Fc Attenuates LPS-Induced Pulmonary Vascular Leakage

**Fig 3:**
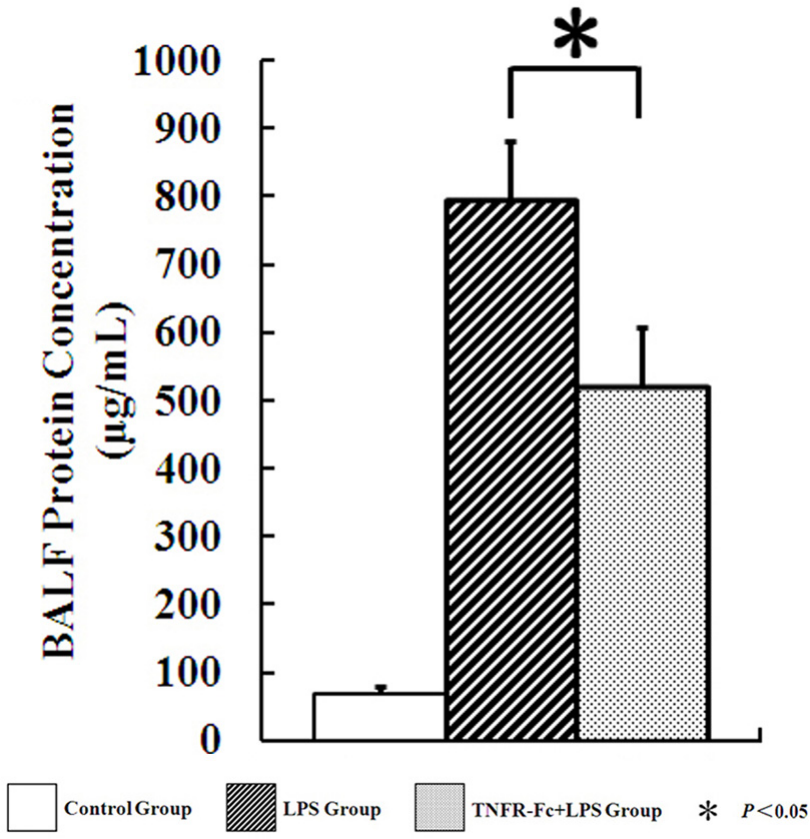
TNFR-Fc produced a beneficial effect for ALI mice. LPS treatment induced pulmonary vascular leakage. Protein concentrations in BALF was noticeably reduced in TNFR-Fc pretreated mice (N = 3 in each group).

BALF protein concentration represents the level of pulmonary vascular leakage, which induced by inflammation. Protein concentrations in BALF increased in LPS group and TNFR-Fc group. LPS administration induces pulmonary vascular leakage as evidenced by increased BALF protein concentration. Pretreatment with TNFR-Fc before LPS administration decreased basal BALF protein levels, compare to LPS group (793.43±87.168, 519.46±88.579, *P <* 0.05) (Fig 3).

### TNFR-Fc Inhibited IKK, I-κB, and NF-κB p65 Phosphorylation in LPS-Induced ALI Mice

Western blot analysis was employed to assess the concentration of phospho-IKK and IκB, which reflects IKK activation and IκB degradation in the lung, both of which are low in unstimulated lungs. After LPS challenge, IKK phosphorylation increased substantially.

**Fig 4:**
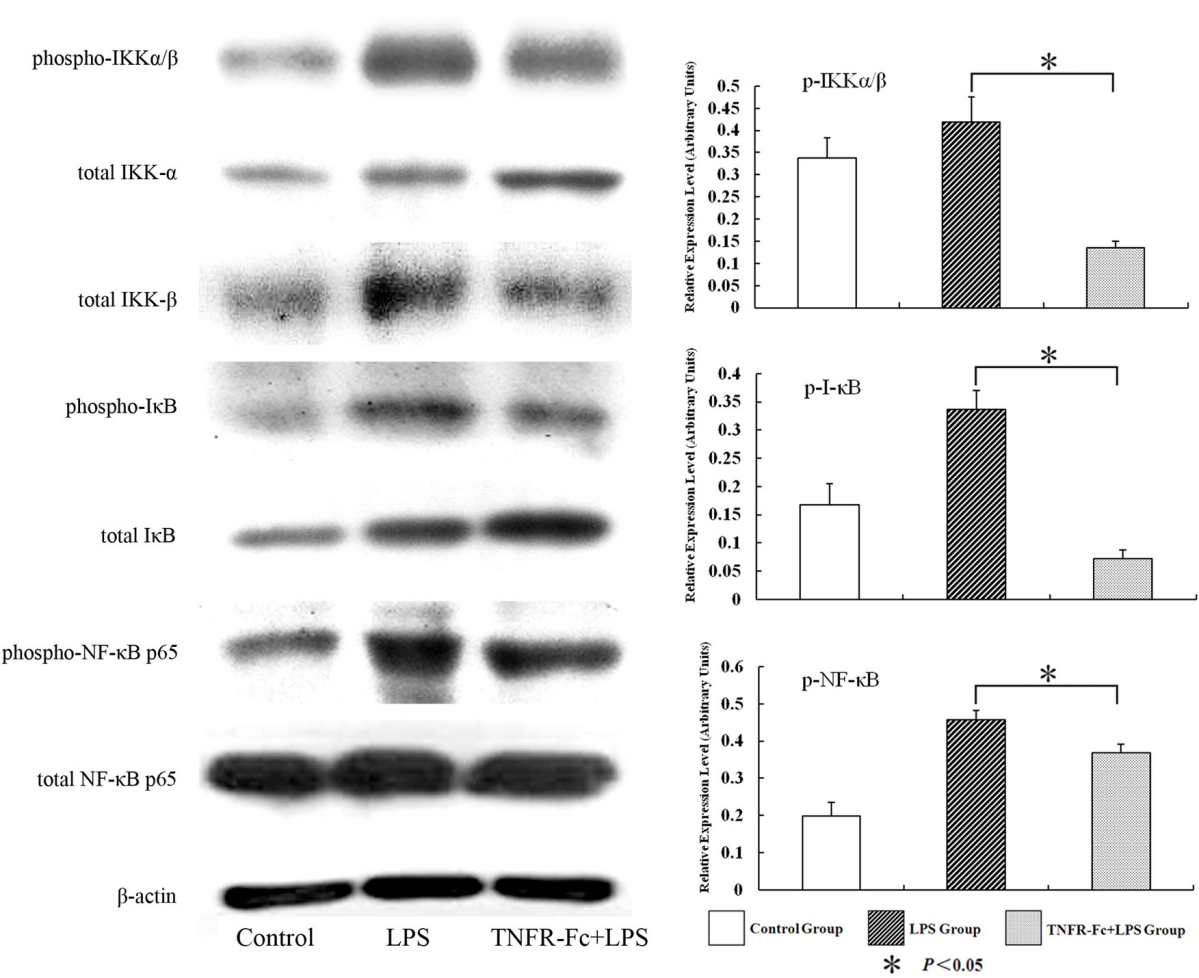
Effect of TNFR-Fc on LPS-induced activation of IKK, IκB, and NF-κB. The relative densities of p-IKK, p-IκB, and p-NF-κB p65 were analyzed by densitometry. All densitometric values were normalized to total protein and are depicted as mean ± SD (N = 3 in each group). Densities of p-IKK, p-IκB, or p-NF-κB p65 in TNRR-Fc + LPS were lower than that in LPS alone (*P <* 0.05).

To investigate the mechanisms underlying TNFR-Fc mediated LPS induction of NF-κB activation, IκB degradation in lung was analyzed by western blot. Notably, LPS treatment increased lung protein content (Fig 4).

### TNF-α Blockage Inhibited NF-κB Binding to TNF-α Promoter and Down-Regulated TNF-α mRNA Expression

**Fig 5:**
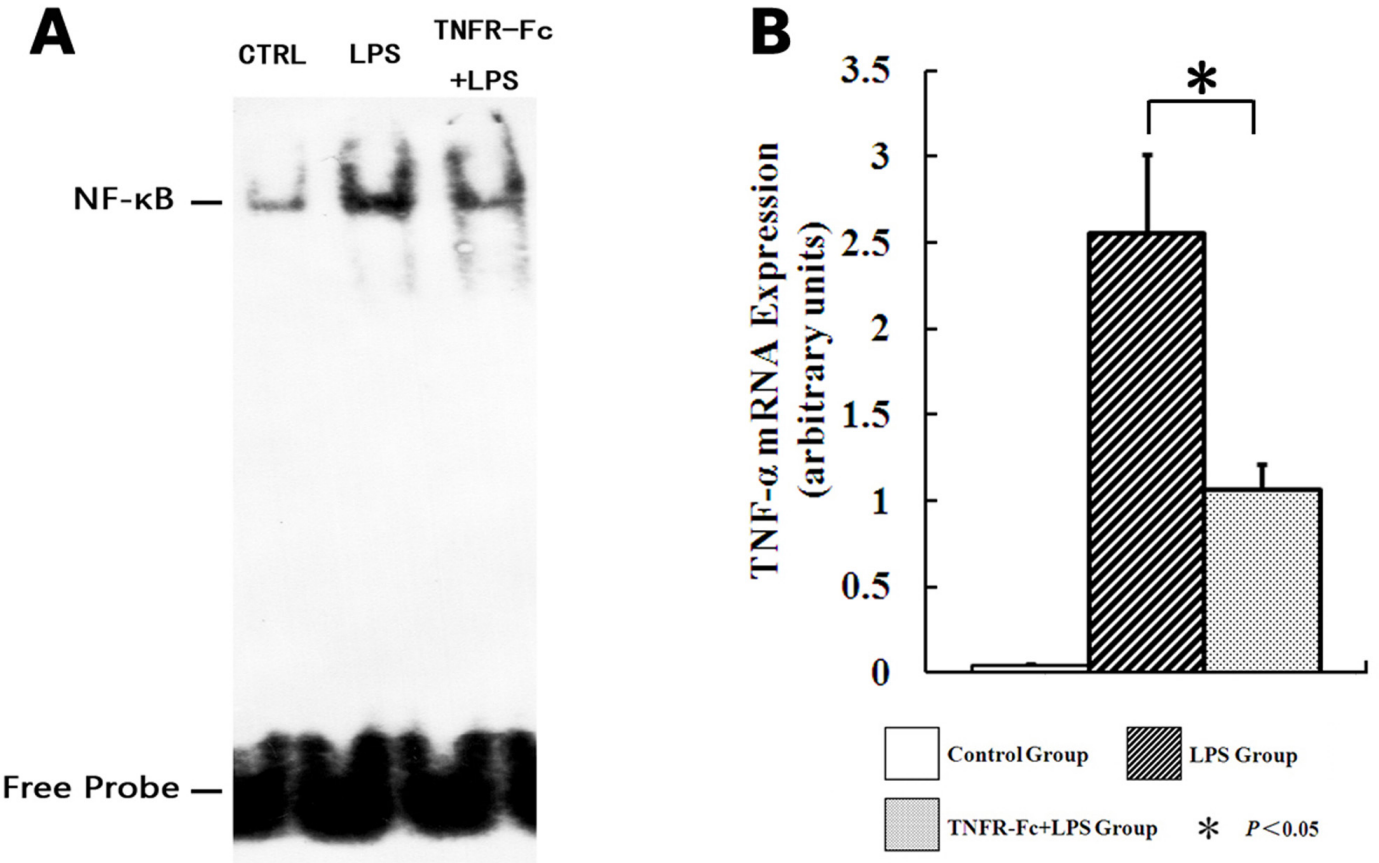
TNFR-Fc pretreatment depresses TNF-α expression via NF-κB feedback. (A)EMSA for NF-κB binding in lung control nuclear extracts, LPS, and TNFR-Fc + LPS mice (N = 3 in each group). LPS administration caused a marked increase in NF-κB binding to biotin-labeled oligonucleotide probes derived from the TNF-α promoter, and TNFR-Fc pretreatment partially inhibited NF-κB binding. (B)qRT-PCR was used to detect TNF-α mRNA expression (N = 3 in each group). LPS challenge raised TNF-α mRNA expression in ALI mice, and TNFR-Fc pretreatment attenuated TNF-α mRNA transcription. Error bars represent SEM. *P < 0.05.

NF-κB is an important transcription factor in TNF-α gene expression. LPS challenge promotes NF-κB nuclear translocation and binding to the TNF-α promoter, followed by TNF-α mRNA expression and pulmonary inflammation. In unstimulated mice, nuclear NF-κB levels were low. Nuclear NF-κB levels increased in LPS treated mice, followed by TNF-α mRNA expression. TNFR-Fc pretreatment prevented LPS-induced nuclear translocation of NF-κB and decreased TNF-α mRNA expression (Fig 5).

### TNF-α Blockage Attenuates Oxidative Damage in LPS-Induced Lung

**Fig 6:**
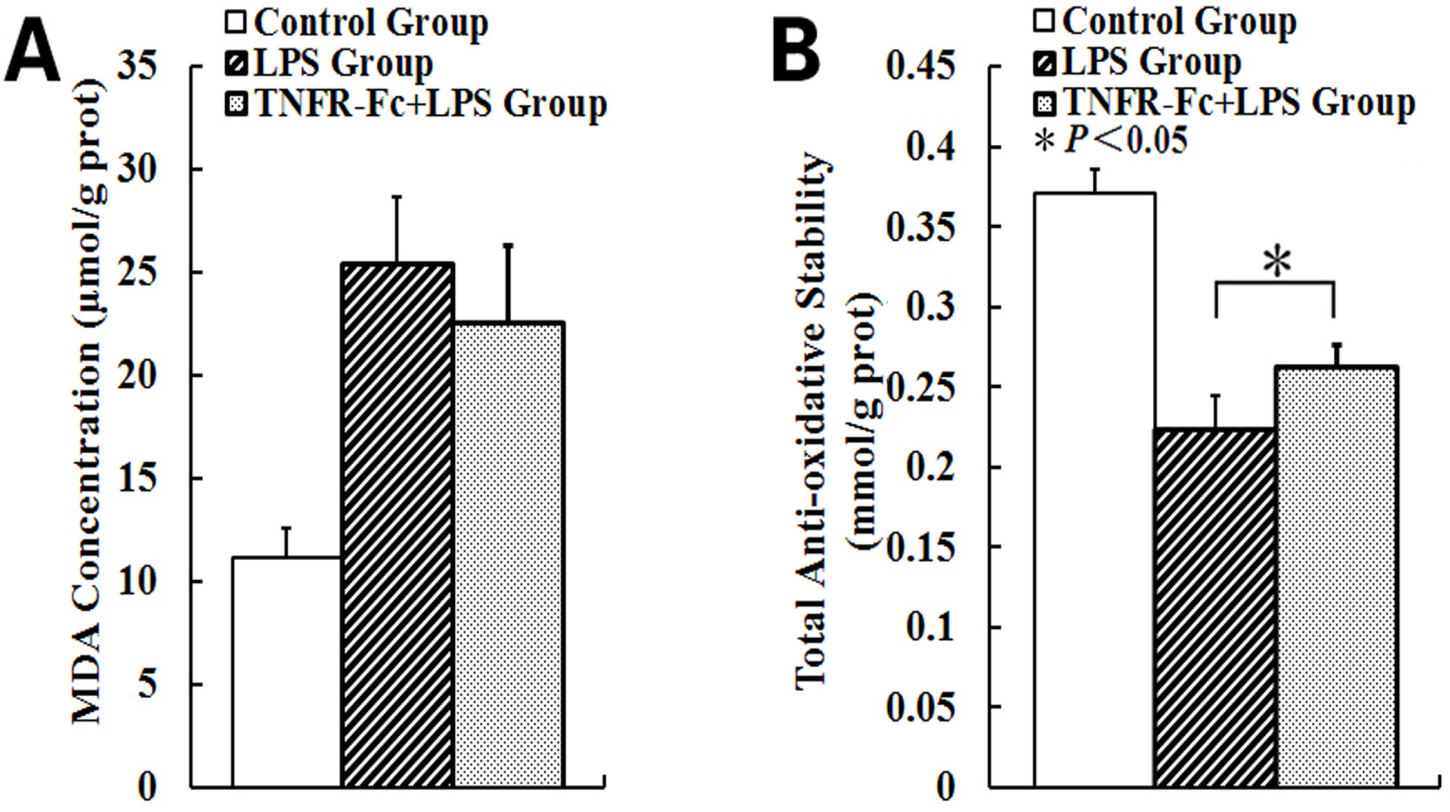
TNFR-Fc attenuated oxidative damage in lung. Mice were challenged by intratracheal LPS administration (N = 3 in each group). TNFR-Fc pretreatment would not decrease MDA concentration compared to LPS group. However, TNFR-Fc pretreatment increased T-AOC after LPS treatment in mice.

Lung MDA concentration, which correlated to oxidative damage, was elevated relative to controls, and increased after LPS challenge. The data showed that TNFR-Fc pretreatment decreased MDA concentration after LPS challenge. However, no difference was observed between LPS group and TNFR-Fc+LPS group. The total anti-oxidative stability in LPS lung, which correlated to inhibiting the oxidative injury, was lower than that in controls, and TNFR-Fc pretreatment raised total anti-oxidative stability after LPS administration (Fig 6).

### TNFR-Fc Down-Regulated Oxidative Enzyme Transcription Levels

**Fig 7:**
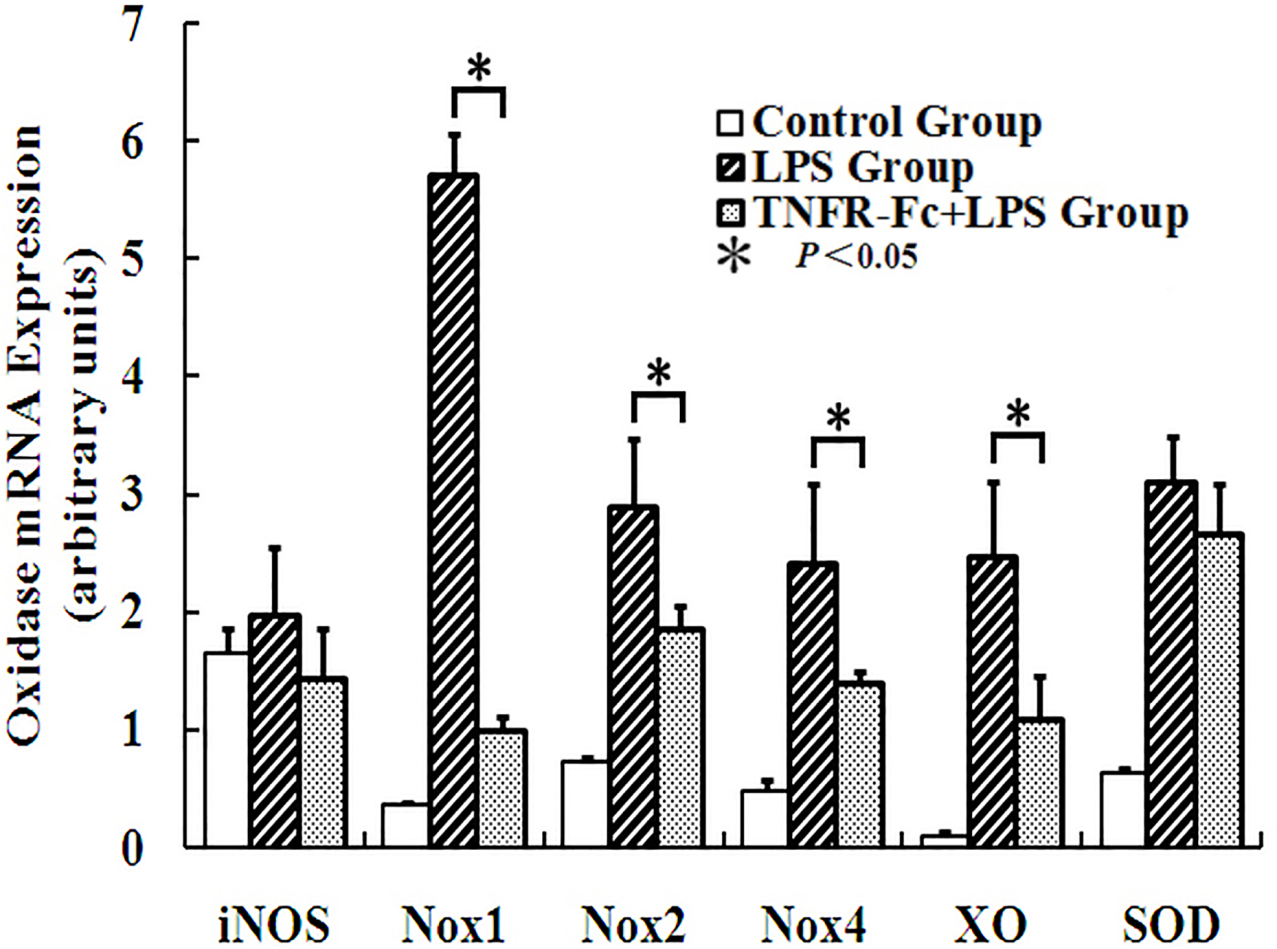
TNFR-Fc diminished transcription levels of Noxs and XO triggered by LPS treatment. TNFR-Fc did not reduce SOD transcription levels after LPS challenge (N = 3 in each group). LPS and TNFR-Fc did not affect iNOS (N = 3 in each group).

Our study explored transcription levels of iNOS, Noxs, XO, and SOD. Except for iNOS, the transcription levels of all oxidative enzymes examined increased after LPS challenge. SOD, an anti-oxidative enzyme, is transcribed concomitantly with other oxidative enzymes. ALI mice pretreated with TNFR-Fc exhibited lower transcription levels of Nox1, Nox2, Nox4, and XO. However, TNFR-Fc did not affect iNOS and SOD transcription in ALI mice (Fig 7).

## Discussion

ALI is a major cause of acute respiratory failure with high morbidity and mortality in critically ill patients [10]. It is characterized by a serious pulmonary inflammatory response resulting in destruction of the microvascular endothelial barrier and protein-rich pulmonary edema. Glucocorticoids benefit ALI patients by inhibiting expression of pro-inflammatory and immune-modulatory genes, such as TNF-α.

TNF-α is a key cytokine in the inflammatory response and is a potential target for therapeutic strategies related to ALI [11, 12], as it is known that patients suffering from an inflammatory response, such as rheumatoid arthritis and Crohn’s disease, will benefit from blocking TNF-α [13,14]. Our study shows that TNFR-Fc targeting TNF-α, and applied intraperitoneally, produces a beneficial effect in an LPS-induced mouse model of ALI, reducing the inflammatory response and lung tissue destruction [15].

In acute or chronic inflammation, a spectrum of cytokines is detected in diseased tissue. As demonstrated by others, LPS-challenged mice accumulate systemic and local inflammation with a significant increase of early pro-inflammatory cytokines (TNF-α and IL-6) in serum or BALF. At the same time, anti-inflammatory cytokines are up-regulated, as well as pro-inflammatory cytokines, and blocking IL-10 augmented TNF-α production two-fold [16]. One might predict that disordered inflammation would improve in ALI when an anti-inflammatory cytokine, such as IL-10, is dominant. Here, BALF and serum TNF-α and IL-6 levels rise after LPS challenge and decrease when mice are pretreated with TNFR-Fc. IL-10 levels rise in ALI mice naturally. However, TNFR-Fc induces BALF IL-10 concentrations. In contrast, we observed increased BALF protein concentrations. Pulmonary vascular leakage suggests an impaired epithelial/endothelial barrier in ALI mice [17] and our in vivo data shows that TNFR-Fc promotes restoration of the endothelial barrier after LPS-induced injury.

We found that TNFR-Fc attenuated these important early stages in ALI pathogenesis. Furthermore, we observed severe lung damage in mice challenged with LPS and TNFR-Fc, preventing change in lung histology. Mice treated with LPS showed serious tissue damage, characterized by interalveolar septal thickening and alveolar wall destruction. Fortunately, TNFR-Fc pretreatment preserved lung parenchymal architecture. Collectively, these observations are in agreement with previous studies indicating that LPS administration induces inflammation, resulting in tissue destruction. Furthermore, TNFR-Fc pretreatment attenuated lung damage in ALI via TNF-α blockage.

An additional mechanism of “inflammatory positive feedback inhibition” of TNF-α blockade was examined in this study. TNF-α is the body’s “fire alarm” and induces rapid arrival of leukocytes from the blood. TNF-α expression induced by microorganism, physical agents, or chemical materials is an important step in the inflammatory cascade [18]. The initial release of TNF-α binds to its receptor, TNFR, and induces proinflammatory cytokine expression (including TNF-α itself), followed by positive feedback. Nevertheless, previous studies show TNF-α gene regulation is associated with NF-κB activation [19, 20].

NF-κB activity is regulated by IκB, which forms a complex with NF-kB in the cytoplasm and inhibits nuclear localization of the dimer [21]. When cells receive signals that activate NF-kB, IKK is phosphorylated and binds to IκB, inducing IκB phosphorylation, followed by IκB degradation through an ubiquitin proteasome pathway [22]. IκB degradation triggers NF-kB translocation from the cytoplasm to the nucleus and activates transcription of specific target genes, such as TNF-α.

In this study, LPS challenge induced IKK and IκB phosphorylation and IκB degradation, increasing NF-κB p65 phosphorylation. Moreover, NF-κB bound specifically to the TNF-α promoter. We also found blocking TNF-α persevered IKK and IκB phosphorylation and inhibited NF-κB nuclear translocation, followed by decreased TNF-α expression. The decreases in NF-κB binding in the TNFR-Fc pretreated mice are consistent with down-regulation of TNF-α and IL-6.

In addition to inflammation, TNF-α triggers oxidative stress in the body. In our previous study, oxidative stress was seen to play an important role in LPS-induced ALI [23]. Both in vitro and in vivo studies have associated TNF-α as an important contributor to oxidative stress, either directly or indirectly. Furthermore, TNF-α modulates the activity and expression of Noxs, a potential source of ROS in lung. The Noxs family has 5 members (Nox1-5), each with homology to the phagocyte catalytic unit gp91phox [24]. Noxs exist in non-phagocytic cells of vascular smooth muscle, endothelium, carotid body, lung, and kidney, and play a role in a variety of events such as oxidative stress, signaling for cell growth or death, oxygen sensing, and inflammatory processes [25]. In this study, Nox1, Nox2, and Nox4 were detected in lung and up-regulated after LPS challenge in ALI mice. TNFR-Fc pretreatment diminished Noxs transcription level, resulting in oxidative damage.

XO is another oxidative enzyme associated with oxidative stress and inflammation [26]. In our study, LPS induced XO transcription in lung. Similar to Noxs, TNFR-Fc inhibited XO expression. SOD is an important oxygen free radical scavenger, which balances oxidative stress. Interestingly, increased SOD transcription levels appeared to compensate for other oxidative enzymes. However, TNFR-Fc did not affect SOD transcription after LPS challenge. Thus, TNFR-Fc pretreatment diminished oxidative damage but did not affect anti-oxidative processes.

## Conclusions

In an intratracheal LPS-induced murine ALI model, TNFR-Fc inhibits the pulmonary inflammatory response consisting of early pro-inflammatory cytokine expression, vascular leakage, and tissue damage of the lung. Therefore, TNF-α blockage breaks cell signaling, including those mediated by MAPKs and NF-κB. Finally, TNF-α blockage inhibited oxidative enzyme expression but did not affect anti-oxidative processes, resulting in diminished oxidative damage. These results suggest that blocking TNF-α may have a beneficial effect on the pulmonary inflammatory response and the oxidative stress in ALI patients. However, it is important to recognize that the current effects occurred when TNFR-Fc was given before LPS administration. Therefore, further investigations are necessary to determine whether similar effects are observed when TNFR-Fc is initiated after the onset of lung injury.

## Supporting Information

S1 Fig TNFR-Fc attenuates lung destruction induced by LPS injection (x200). Representative hematoxylin and eosin-stained preparations of lung tissue from mice 2 h after LPS/saline treatment (N = 3 in each group). (A) Saline-treated mice display regular lung histology. (B) LPS-treated mice displayed typical signs of congestion and disruption of alveolar architecture. (C) TNFR-Fc-pretreated ALI mice were largely protected from these alterations. (D)Histological assessment of the effect TNFR-Fc on LPS-induced lung inflammation and injury (N = 3 in each group). TNFR-Fc pretreatment decreased the ALI score in ALI mice.
(TIF)

S2 Fig Concentration of TNF-α, IL-6, and IL-10 in serum and BALF after LPS/saline injection. Increase in TNF-α and IL-6 from baseline in serum/BALF obtained from mice in response to LPS or TNFR-Fc + LPS. TNFR-Fc decreased serum/BALF TNF-α and IL-6 levels in ALI mice. Serum IL-10 concentration increased after LPS injection. TNFR-Fc increased serum IL-10 in ALI mice. (A) TNF-α, (B) IL-6, (C) IL-10. Error bars represent SEM (N = 3 in each group). *P < 0.05.
(TIF)

S3 Fig TNFR-Fc produced a beneficial effect for ALI mice. LPS treatment induced pulmonary vascular leakage. Protein concentrations in BALF was noticeably reduced in TNFR-Fc pretreated mice (N = 3 in each group).
(TIF)

S4 Fig Effect of TNFR-Fc on LPS-induced activation of IKK, IκB, and NF-κB. The relative densities of p-IKK, p-IκB, and p-NF-κB p65 were analyzed by densitometry. All densitometric values were normalized to total protein and are depicted as mean ± SD (N = 3 in each group). Densities of p-IKK, p-IκB, or p-NF-κB p65 in TNRR-Fc + LPS were lower than that in LPS alone (*P <* 0.05).
(TIF)

S5 Fig TNFR-Fc pretreatment depresses TNF-α expression via NF-κB feedback. (A)EMSA for NF-κB binding in lung control nuclear extracts, LPS, and TNFR-Fc + LPS mice (N = 3 in each group). LPS administration caused a marked increase in NF-κB binding to biotin-labeled oligonucleotide probes derived from the TNF-α promoter, and TNFR-Fc pretreatment partially inhibited NF-κB binding. (B)qRT-PCR was used to detect TNF-α mRNA expression (N = 3 in each group). LPS challenge raised TNF-α mRNA expression in ALI mice, and TNFR-Fc pretreatment attenuated TNF-α mRNA transcription. Error bars represent SEM. *P < 0.05.
(TIF)

S6 Fig TNFR-Fc attenuated oxidative damage in lung. Mice were challenged by intratracheal LPS administration (N = 3 in each group). TNFR-Fc pretreatment would not decrease MDA concentration compared to LPS group. However, TNFR-Fc pretreatment increased T-AOC after LPS treatment in mice.
(TIF)

S7 Fig TNFR-Fc diminished transcription levels of Noxs and XO triggered by LPS treatment. TNFR-Fc did not reduce SOD transcription levels after LPS challenge (N = 3 in each group). LPS and TNFR-Fc did not affect iNOS (N = 3 in each group).
(TIF)

S1 Table PCR primers and their associated annealing temperatures. Annealing temperature dictated by the other genes being amplified in the same reaction.
(DOC)
