# Supplementary figures and images for: Inhibition of Acute Lung Injury by TNFR-Fc through Regulation of an Inflammation-Oxidative Stress Pathway
Source: PLoS One. 2016 Mar 18;11(3):e0151672. doi: 10.1371/journal.pone.0151672 (PMC4798551; doi:10.1371/journal.pone.0151672)

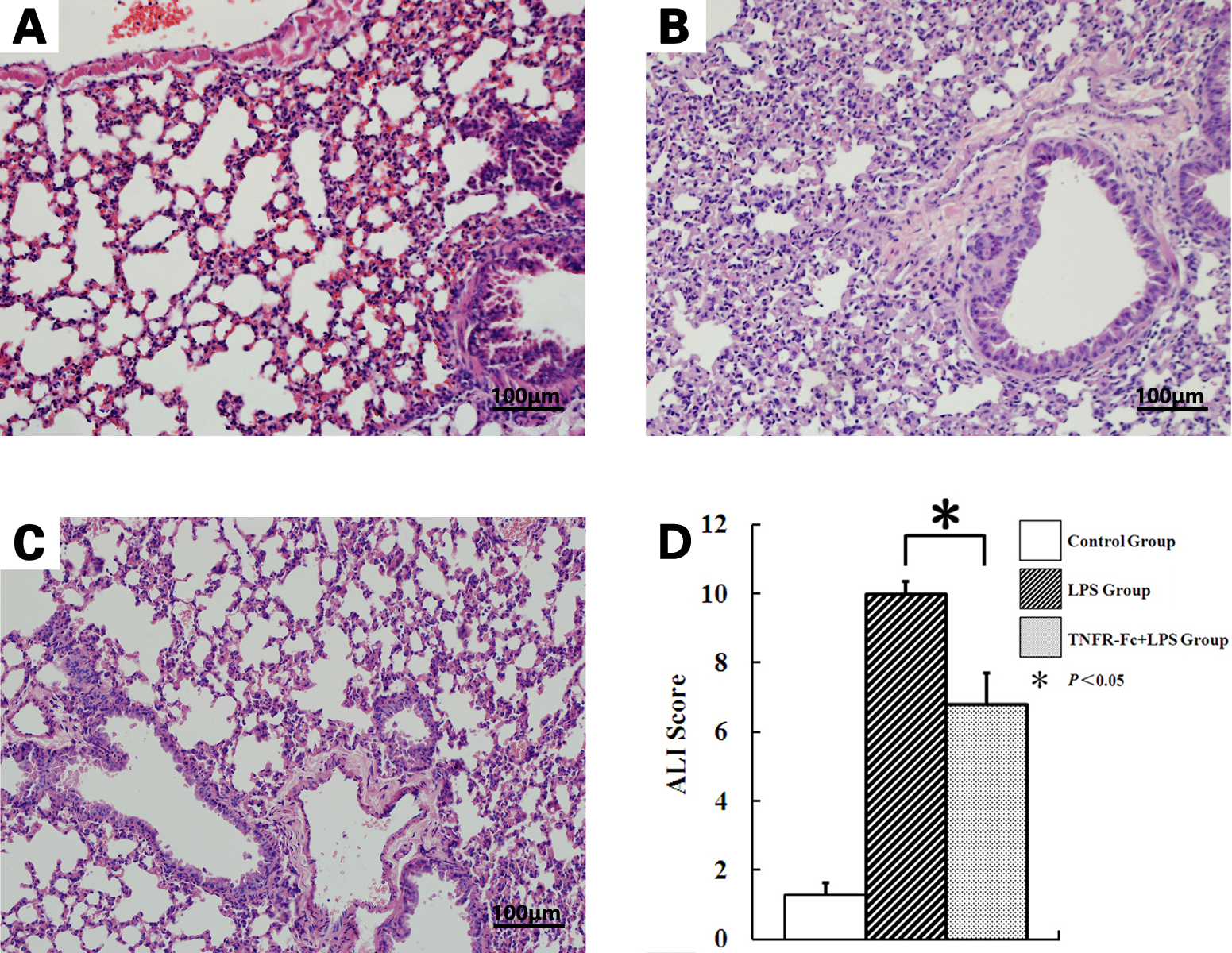

Supplement: S1 Fig — Representative hematoxylin and eosin-stained preparations of lung tissue from mice 2 h after LPS/saline treatment (N = 3 in each group). (A) Saline-treated mice display regular lung histology. (B) LPS-treated mice displayed typical signs of congestion and disruption of alveolar architecture. (C) TNFR-Fc-pretreated ALI mice were largely protected from these alterations. (D)Histological assessment of the effect TNFR-Fc on LPS-induced lung inflammation and injury (N = 3 in each group). TNFR-Fc pretreatment decreased the ALI score in ALI mice. (TIF) [file pone.0151672.s001.tif]

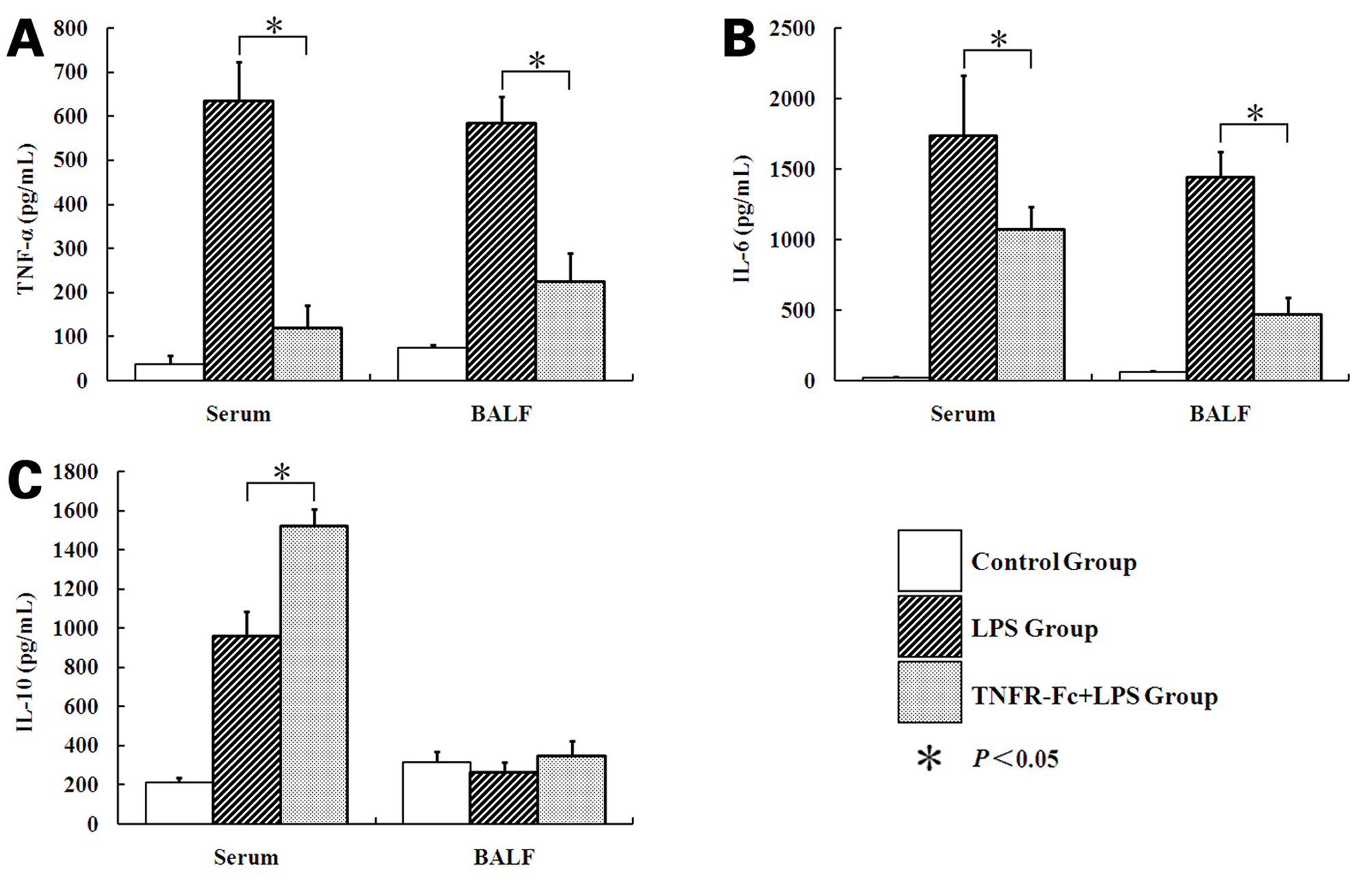

Supplement: S2 Fig — Increase in TNF-α and IL-6 from baseline in serum/BALF obtained from mice in response to LPS or TNFR-Fc + LPS. TNFR-Fc decreased serum/BALF TNF-α and IL-6 levels in ALI mice. Serum IL-10 concentration increased after LPS injection. TNFR-Fc increased serum IL-10 in ALI mice. (A) TNF-α, (B) IL-6, (C) IL-10. Error bars represent SEM (N = 3 in each group). *P < 0.05. (TIF) [file pone.0151672.s002.tif]

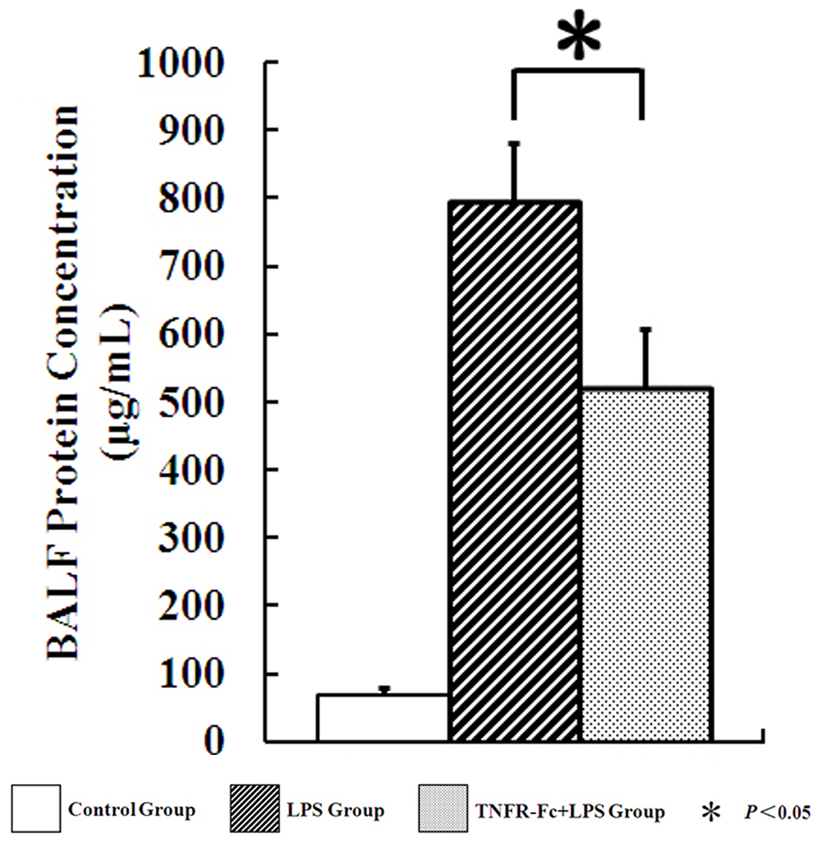

Supplement: S3 Fig — LPS treatment induced pulmonary vascular leakage. Protein concentrations in BALF was noticeably reduced in TNFR-Fc pretreated mice (N = 3 in each group). (TIF) [file pone.0151672.s003.tif]

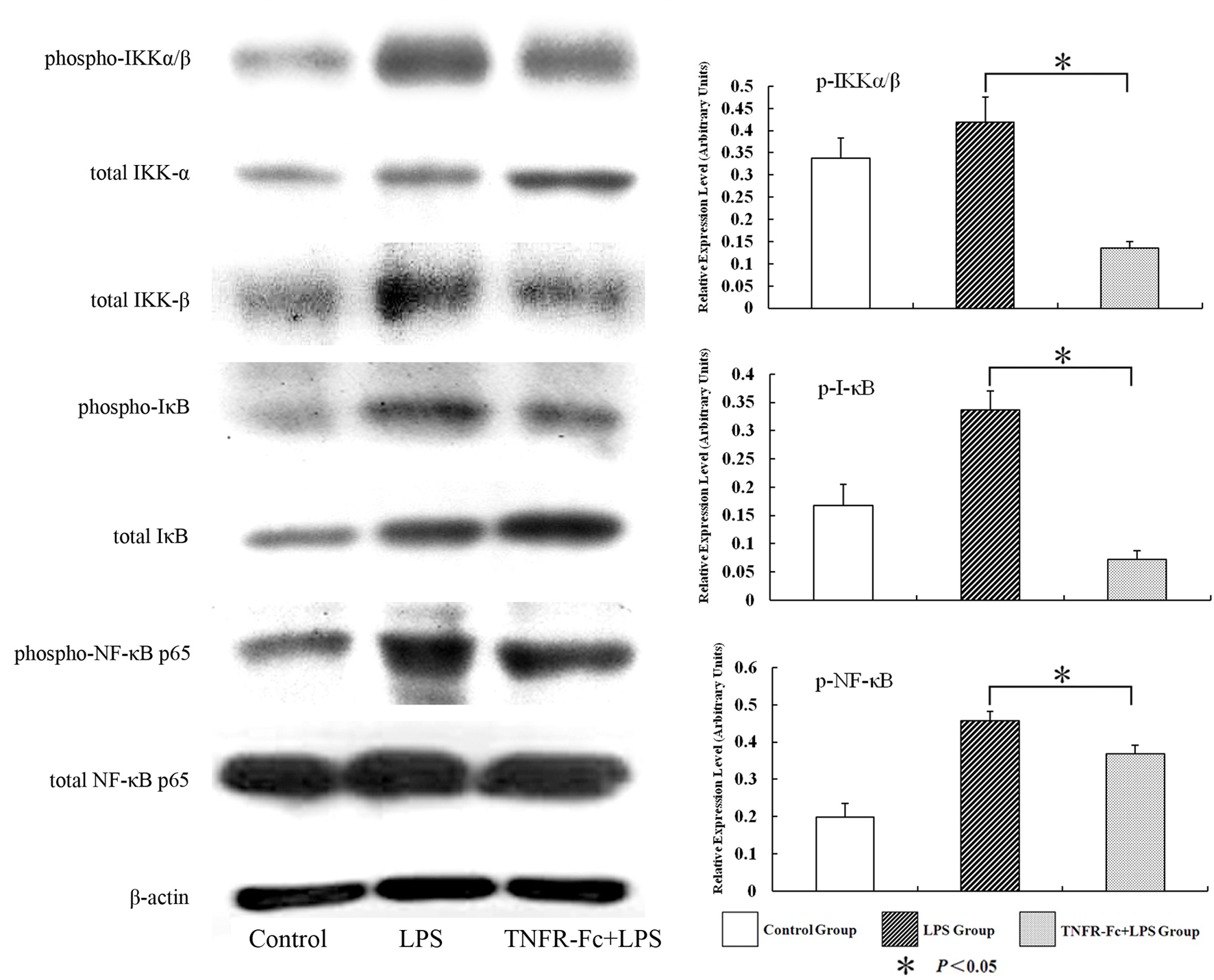

Supplement: S4 Fig — The relative densities of p-IKK, p-IκB, and p-NF-κB p65 were analyzed by densitometry. All densitometric values were normalized to total protein and are depicted as mean ± SD (N = 3 in each group). Densities of p-IKK, p-IκB, or p-NF-κB p65 in TNRR-Fc + LPS were lower than that in LPS alone (P < 0.05). (TIF) [file pone.0151672.s004.tif]

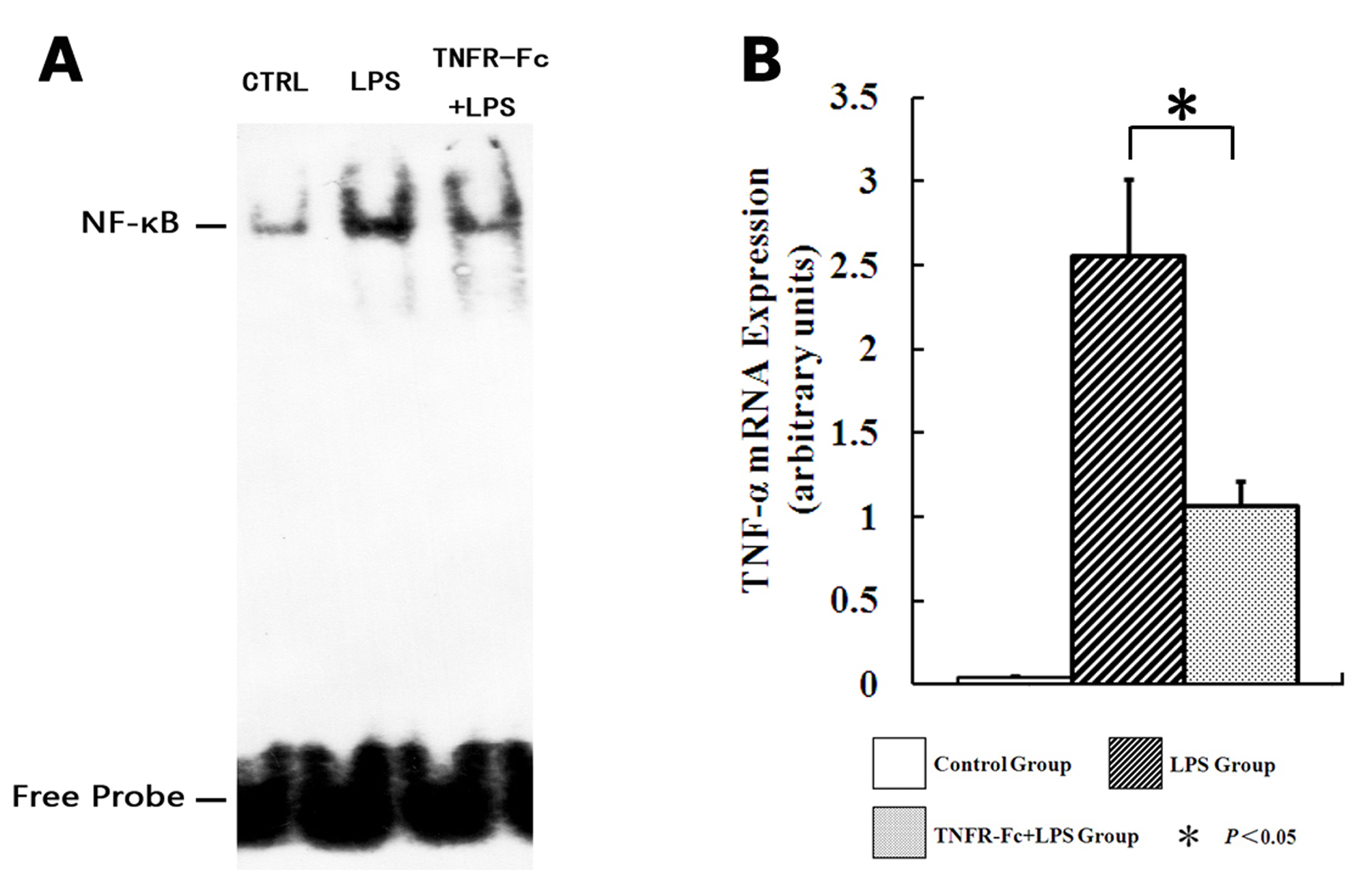

Supplement: S5 Fig — (A)EMSA for NF-κB binding in lung control nuclear extracts, LPS, and TNFR-Fc + LPS mice (N = 3 in each group). LPS administration caused a marked increase in NF-κB binding to biotin-labeled oligonucleotide probes derived from the TNF-α promoter, and TNFR-Fc pretreatment partially inhibited NF-κB binding. (B)qRT-PCR was used to detect TNF-α mRNA expression (N = 3 in each group). LPS challenge raised TNF-α mRNA expression in ALI mice, and TNFR-Fc pretreatment attenuated TNF-α mRNA transcription. Error bars represent SEM. *P < 0.05. (TIF) [file pone.0151672.s005.tif]

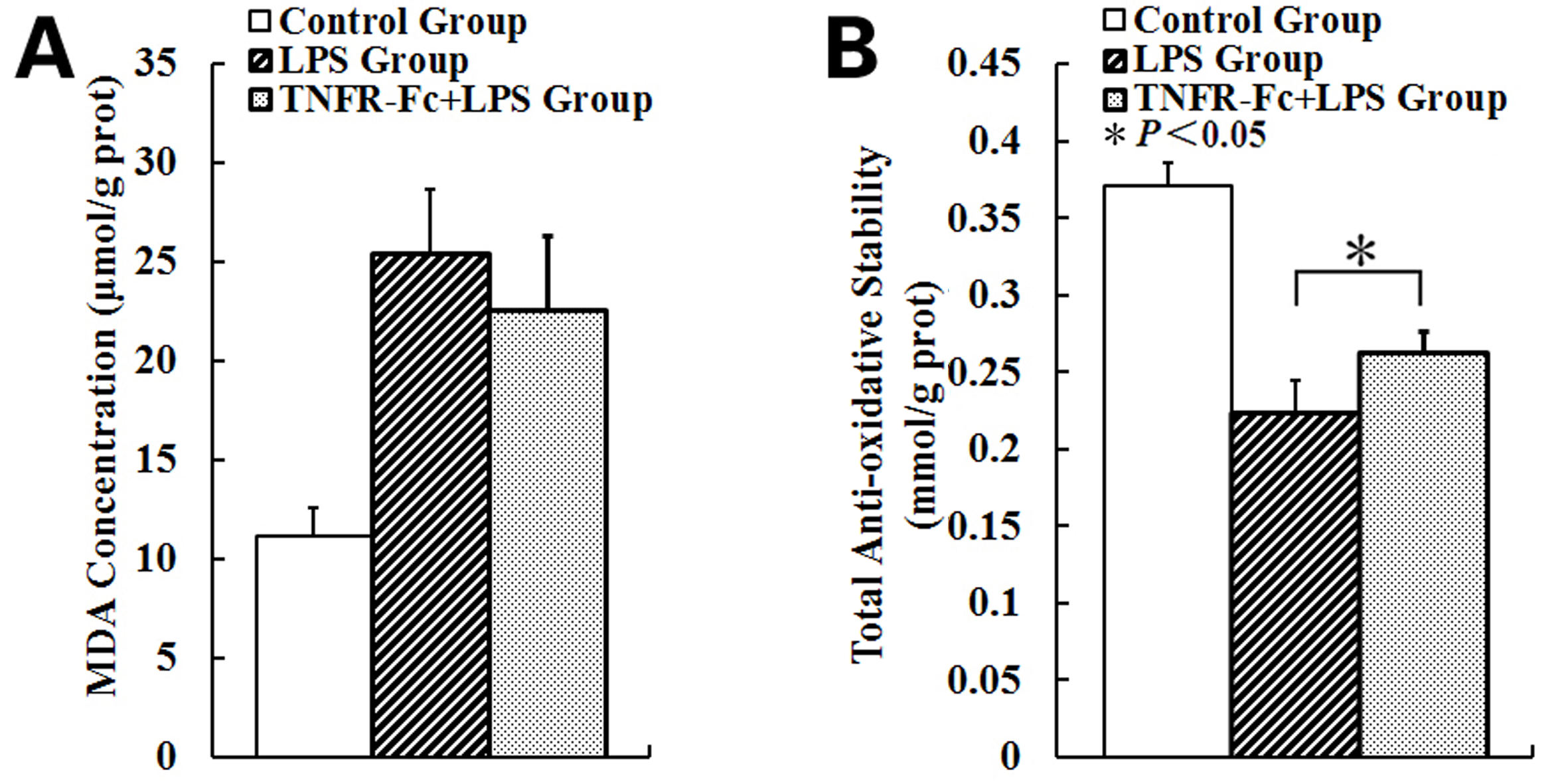

Supplement: S6 Fig — Mice were challenged by intratracheal LPS administration (N = 3 in each group). TNFR-Fc pretreatment would not decrease MDA concentration compared to LPS group. However, TNFR-Fc pretreatment increased T-AOC after LPS treatment in mice. (TIF) [file pone.0151672.s006.tif]

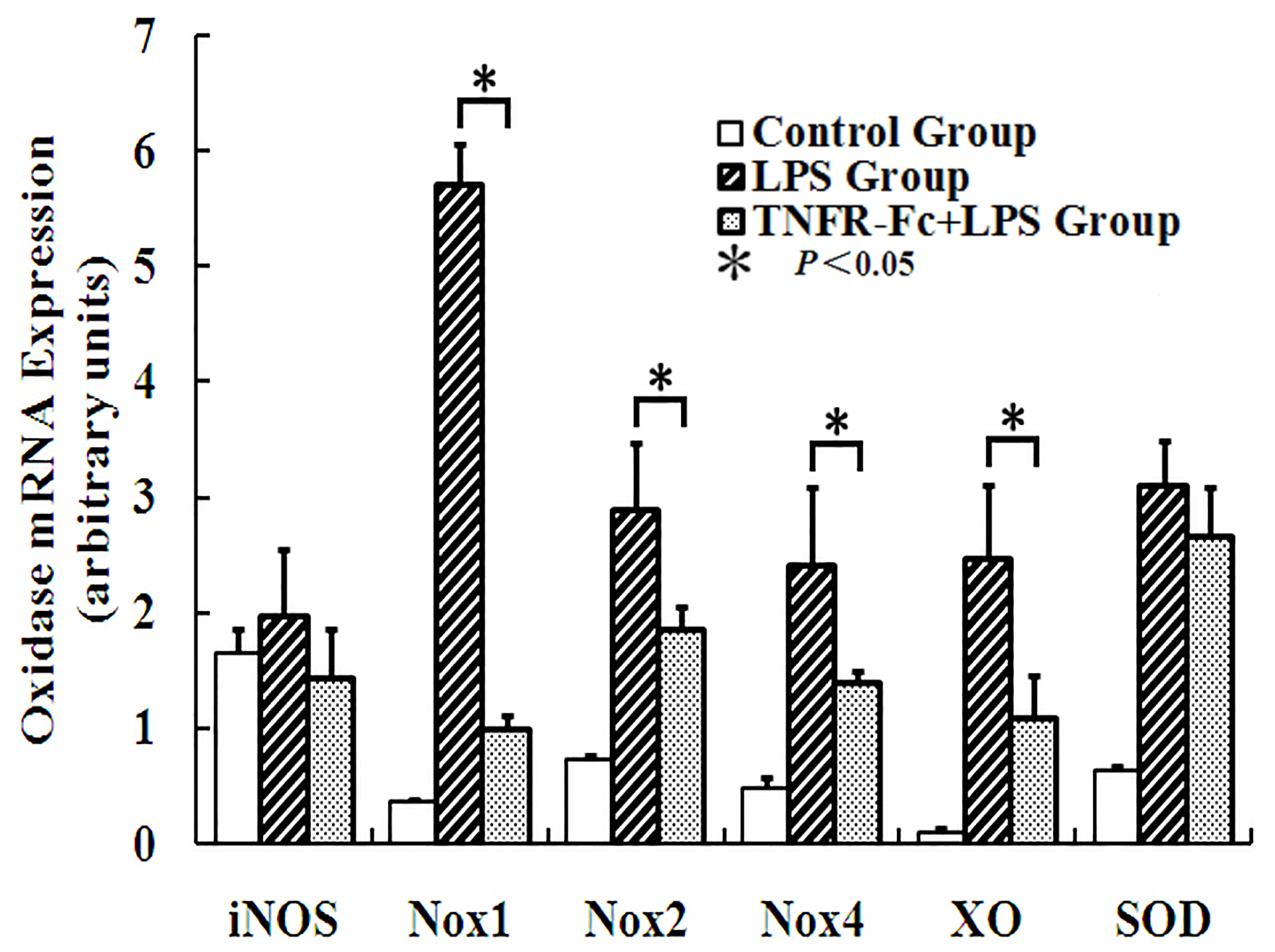

Supplement: S7 Fig — TNFR-Fc did not reduce SOD transcription levels after LPS challenge (N = 3 in each group). LPS and TNFR-Fc did not affect iNOS (N = 3 in each group). (TIF) [file pone.0151672.s007.tif]
