# Supplementary material for: Inhibition of Acute Lung Injury by TNFR-Fc through Regulation of an Inflammation-Oxidative Stress Pathway
Source: PLoS One. 2016 Mar 18;11(3):e0151672. doi: 10.1371/journal.pone.0151672 (PMC4798551; doi:10.1371/journal.pone.0151672)
Supplement: S1 Table — Annealing temperature dictated by the other genes being amplified in the same reaction. (DOC) [file pone.0151672.s008.doc]

Table 1. PCR primers and their associated annealing temperatures.

| **Gene** | **Oligonucleotide primers（5’→3’）** | **Annealing temperature (°C)** |
| --- | --- | --- |
| **TNF-α** | TTCTGTCCCTTTCACTCACTGG | 55 |
|  | TTGGTGGTTTGCTACGACGTGG |  |
| **iNOS** | CACATTCAGATCCCGAAACGC | 59 |
|  | CAATCCACAACTCGCTCCAAGA |  |
| **Nox1** | TGGCATCCCTTCACTCTGA | 52 |
|  | GGCACGCTGGAATTTGTAC |  |
| **Nox2** | CCCTCCTATGACTTGGAAATG | 53 |
|  | TCCGTCCAGTCTCCCACAATA |  |
| **Nox4** | AGACAAATGTAGACACTCACC | 49 |
|  | CACAATAAAGGCACAAAGGT |  |
| **XO** | TGGCTTGCTCAGAAGTAGA | 53 |
|  | CCTCACGGACCAGGATTTAC |  |
| **SOD** | AGTTCAATGGTGGTGGTCATA | 53 |
|  | CAATC CCCAGCAGTGGAATAA |  |
| **β-actin** | AGGGAAATCGTGCGTGACATCAAA | * |
|  | ACTCATCGTACTCCTGCTTGCTGA |  |

* Annealing temperature dictated by the other genes being amplified in the same reaction.
